# Correlation analysis between HLA-DQA1*0102/DQB1*0602 genotypes and narcolepsy patients in China

**DOI:** 10.3389/fneur.2024.1379723

**Published:** 2024-04-25

**Authors:** Wanyu Zhao, Baokun Zhang, Zian Yan, Mengke Zhao, Xiao Zhang, Xiaoyu Zhang, Xiaomin Liu, Jiyou Tang

**Affiliations:** ^1^Department of Neurology, The First Affiliated Hospital of Shandong First Medical University, Jinan, Shandong, China; ^2^Department of Neurology, Shandong Qianfoshan Hospital, Cheeloo College of Medicine, Shandong University, Jinan, Shandong, China

**Keywords:** narcolepsy, human leukocyte antigen, orexin-A, multiple sleep latency test, cataplexy, EDS

## Abstract

**Background and objective:**

At present, the etiology of narcolepsy is not fully understood, and it is generally believed to be an autoimmune reaction caused by interactions between environmental and genetic factors. Human leukocyte antigen (HLA) class II genes are strongly associated with this gene, especially HLA-DQB1*0602/DQA1*0102. In this study, we mainly analyzed the correlation between different genotypes of HLA-DQB1*0602/DQA1*0102 and clinical manifestations in Chinese patients with narcolepsy.

**Experimental method:**

Narcolepsy patients who were treated at the Department of Neurology, The First Affiliated Hospital of Shandong First Medical University from January 2021 to September 2023 were selected. General information, sleep monitoring data, cerebrospinal fluid (CSF) orexin levels, and human leukocyte antigen gene typing data were collected. The statistical analysis was performed using SPSS 26.0, and the graphs were drawn using GraphPad Prism 9.5.

**Main results:**

A total of 78 patients were included in this study. The DQA1 and DQB1 gene loci were detected in 54 patients, and only the DQB1 gene locus was detected in 24 narcoleptic patients. The most common allele at the HLA-DQB1 locus was *0602 (89.7%), and the most common genotype at this locus was *0602*0301 (19.2%), followed by *0602*0602 (17.9%). The most common phenotype of the HLA-DQA1 locus is *0102 (92.6%), and the most common genotype of this locus is *0102*0102 (27.8%), followed by *0102*0505 (14.8%). There were significant differences (*p* < 0.05) between HLA-DQB1*0602-positive and HLA-DQB1*0602-negative patients in terms of orexin-A levels, presence or absence of cataplexy, UNS, PSG sleep latency, REM sleep latency, N1 sleep percentage, oxygen depletion index, and average REM latency on the MSLT. The HLA-DQA1*0102-positive and HLA-DQA1*0102-negative patients showed significant differences (*p* < 0.05) in disease course, presence or absence of sudden onset, PSG REM sleep latency, N1 sleep percentage, and average REM latency on the MSLT. There were significant differences in the average REM latency of the MSLT between HLA-DQB1*0602/DQA1*0102 homozygous and heterozygous patients *p* < 0.05, and no differences were found in the baseline data, orexin-A levels, scale scores, or other sleep parameters.

**Conclusion:**

Different genotypes of HLA-DQA1*0102/DQB1*0602 are associated with symptoms of cataplexy in Chinese narcoleptic patients. Homozygous individuals have a shorter mean REM latency in the MSLT, greater genetic susceptibility, and relatively more severe sleepiness.

## Introduction

Narcolepsy is a central sleep disorder characterized by excessive daytime sleepiness (EDS), cataplexy, hypnagogic hallucinations, sleep paralysis, and nocturnal sleep disturbance (1). Narcolepsy can be divided into type 1 narcolepsy (NT1) and type 2 narcolepsy (NT2) based on the absence of hypothalamic orexins (2). The current cause of narcolepsy is unknown; it is generally believed to be an autoimmune reaction caused by the interaction of environmental factors and genetic factors, leading to irreversible loss of HCRT neurons (3). However, the latest research by Tafti et al. revealed that the HCRT neurons in narcolepsy patients are not destroyed but are only inactive epigenetically. If the HCRT gene can be reactivated, narcolepsy can be treated or even cured (4). Approximately 8–10% of patients have a family history, and the incidence of first-generation direct relatives of NT1 patients is 20–70 times greater than that of the general population. Latorre et al. used *in vitro* antigen stimulation and sensitive T-cell library screening methods and found that all NT1 patients and some NT2 patients had HCRT-specific memory CD4+ T cells and CD8+ T cells in their blood, while healthy control individuals did not. This finding reinforces the autoimmune etiology of narcolepsy (5). HLA-DQ molecules are receptor proteins located on the surface of antigen-presenting cells and are MHC II 12 heterodimers; they participate in the selection of T-cell libraries in the thymus and play a core role in the adaptive immune response by providing peptides to CD4+ T lymphocyte antigen receptors (6).

Episodic sleeping sickness is strongly correlated with human leukocyte antigen (HLA) class II genes, and mutations in the HLA complex increase the risk of autoimmune reactions in orexinergic neurons in the brain (7), especially the DRB1*1501/DRB5*0101/DQA1*0102/DQB1*0602 haplotype, while the DRB1*0701/DRB4*01/DQA1*0201/DQB1*02 haplotype may have a slight protective effect on this disease (8).

The dimerization of the α and β strands of HLA gene chromosomes results in the formation of four different functional HLA-DQ molecules. Due to the high affinity between DQA1*0102 and DQBI*0602, there is a strong linkage imbalance, and their DQα and DQβ chains are easily bound to form HLA-DQ dimers, which are susceptible to narcolepsy (9). DQB1*0602 can increase the risk of NT1 by 200 times (10), and DQA1*0102, a homologous allele, also has a similar risk (11). In various races (Japan, South Korea, Caucasians, African Americans), this disease is closely related to the HLA-DQ dimer (12–15). The positive rate of DQB1*0602 in typical Chinese patients is as high as 95%, and many DQA1*0102 haplotypes do not carry DQB1*0602. It can be predicted through the DQA1/DQB1 allele competition model that DQA1*0102 has a certain impact on the susceptibility of DQA1*0102/DQB1*0602 (16). Related studies have shown that the relative risk of carrying DQA1*0102 is approximately 1/2 of the risk of DQB1*0602 (8).

Therefore, we speculated that patients with Chinese narcolepsy who carry HLADQA1*0102 and DQB1*0602 may have different severities of symptoms and clinical manifestations. Homozygous and heterozygous genes may differ in sex, age of onset, disease course, scale scores, and sleep monitoring data. To verify the above hypothesis, we conducted the present study.

## Research objects and methods

### Patients

This study included a total of 78 patients who were diagnosed with narcolepsy at the Department of Neurology, the First Affiliated Hospital of Shandong First Medical University, from November 2021 to March 2023. According to the International Classification of Sleep Disorders (ICSD-3) (17), 78 patients with narcolepsy were enrolled in the study and divided into two groups: NT1 (*n* = 56) and NT2 (*n* = 22). As this study mainly analyzed the correlation between genetic susceptibility genes and patients with narcolepsy, no normal control group was used. The patients were mainly Han Chinese, all from northern China, aged between 11 and 68 years. In addition to demographic data, all patients completed subjective scale evaluations, polysomnography, multiple sleep latency tests, lumbar puncture surgery, HLA gene testing, and recorded data. The study was conducted in accordance with the principles expressed in the Helsinki Declaration, and each participant signed an informed consent form, which was reviewed and approved by the Ethics Committee of the First Affiliated Hospital of Shandong First Medical University.

### Scale assessment

The patients were evaluated by a qualified and clinically experienced doctor using a scale that mainly observes the patient’s subjective feelings about their nighttime sleep quality, daytime sleepiness, depression, and anxiety levels, as well as consistency with objective examination parameters, and excludes sleep disorders caused by sleep deprivation, insomnia, and mental illness. The main scales used were the Pittsburgh Sleep Quality Index (PSQI), Epowers Sleepiness Scale (ESS), Ullanlinna Narcolepsy Scale (UNS), Hamilton Depression Rating Scale (HAMD), and Hamilton Anxiety Rating Scale (HAMA).

### Objective evaluation

After the subjective assessments of the subjects, sleep data were collected by sleep center physicians, mainly through two electrophysiological examinations: standard polysomnography (PSG) and multiple sleep latency tests (MSLTs). The PSG is an important reference or gold standard for evaluating nighttime sleep. The MSLT is a widely used diagnostic tool for narcolepsy and idiopathic hypersomnia. The monitoring was conducted in our sleep medicine center. The monitoring equipment used was the Australian Conti Greal series polysomnography monitor. After monitoring, according to the standards of the American Academy of Sleep Medicine Sleep and Related Events Interpretation Manual Rules, Terminology, and Technical Specifications (AASM 2.3 edition), experienced physicians at our sleep medicine center interpreted the data using ProFusion Sleep3 analysis (18, 19).

### Orexin-A levels in CSF

CSF orexin-A levels were measured in three batches by the Gold Domain Laboratory of Radioimmunoassay (located at No. 10, Spiral 3 Road, International Biological Island, Guangzhou, Guangdong Province). CSF orexin-A levels were determined by radioimmunoassay at the Stanford University Narcolepsy Center. The difference between different batches was 2.13%, and the acceptable standard was less than 1/3TEA (maximum allowable error): ± 10%. A detection level less than 110 pg./mL indicates type 1 narcolepsy, 110 pg./mL to 200 pg./mL indicates mild abnormalities, and a normal concentration greater than 200 pg./mL indicates 60% sensitivity, 98% specificity, and 94% positive predictive value (20).

### HLA typing

HLA genotyping was conducted by a laboratory accredited by the First Affiliated Hospital of Shandong First Medical University. All groups of patients were genotyped using second-generation sequencing (NGS) at a high-resolution level, and exons 2 and 3 were sequenced at the HLADQA1 and HLADQB1 loci.

### Statistical analysis

The data were analyzed using the SPSS 26.0 system. Nonparametric tests were conducted on the HLA allele frequency and haplotype frequency of the subjects, as well as their clinical characteristics, demographic data, scale scores, sleep monitoring data, and orexin-A levels. Differences were considered to be statistically significant at *p* < 0.05. We used the GraphPad Prism 9.5 system to plot data with significant differences in a block diagram.

## Results

A total of 78 patients were diagnosed with narcolepsy, of whom 50 (64.1%) were male and 28 (35.9%) were female, with a median age at diagnosis of 11–68 years (median of 18 years). Fifty-six patients (71.8%) were classified as NT1, 22 patients (28.2%) were classified as NT2, 78 patients (100%) had daytime drowsiness and a course of illness of 3–510 months (median 48 months), 51 patients (65.4%) had sudden onset, 26 patients (33.3%) had sleep paralysis, and 26 patients (33.3%) had hallucinations from falling asleep (Table 1).

**Table 1:** Demographic characteristics of narcolepsy patients.

	NT1 (*n* = 56)	NT2 (*n* = 22)	Total (*n* = 78)	*p*-value^*^
Gender (male), *N* (%)	34 (61.8%)	16 (69.6%)	50 (64.1%)	ns^a^
Age, median (range), yr	19 (16 ~ 29)	17 (15 ~ 21)	18 (16 ~ 29)	ns^b^
Age of onset, median (range), yr	14 (11 ~ 19)	14 (12 ~ 16)	14(11 ~ 18)	ns^b^
The duration of the disease, median (range), month	51 (24 ~ 96)	36 (12 ~ 84)	48 (24 ~ 84)	ns^b^
BMI, median (range)	27.45 (25.295 ~ 30.93)	24.72 (19.8975 ~ 27.4725)	27.2 (23.34 ~ 30)	0.006^b^
Qrexin-A, median (range), pg./ml	16.8 (11.3 ~ 33)	210.6 (194.3 ~ 231.7)	21.2 (14.1 ~ 120)	0.000^a^
Accompanied by cataplexy, *N* (%)	51 (91.1%)	0	51 (65.4%)	0.000^a^
Accompanied by hypnagogic hallucinations, *N* (%)	20 (35.7%)	6 (27.3%)	26 (33.3%)	ns^a^
Accompanied by sleep paralysis, *N* (%)	22 (39.3%)	4 (18.2%)	26 (33.3%)	ns^a^

a χ2 test.

b Mann–Whitney U test.

Fifty-four patients diagnosed with narcolepsy were tested for two gene loci, DQA1 and DQB1, while 24 patients were only tested for the DQB1 gene locus. There were 11 cases (19.6%) of HLA-DQB1*0602 homozygosity in the NT1 population and 12 cases (30%) of HLA-DQA1*0102 homozygosity. In the NT2 population, there were 3 homozygous cases (13.6%) of HLA-DQB1*0602 and 3 homozygous cases (21.4%) of HLA-DQA1*0102. The most common allele at the HLA-DQB1 locus is DQB1*0602 (89.7%), and the most common genotype at this locus is DQB1*0602/*0301 (19.2%), followed by DQB1*0602/*0602 (17.9%). The most common phenotype at the HLA-DQA1 locus was DQA1*0102 (92.6%), and the most common genotype at this locus was DQA1 0102/*0102 (27.8%), followed by DQA1*0102/*0505 (14.8%) (Table 2).

**Table 2:** Observed phenotypic frequencies for HLA-DQB1 and HLA-DQA1 alleles and genetic associations of narcolepsy patients.

HLA-DQB1	NT1 (*n* = 56)	NT2 (*n* = 22)	Total (*n* = 78)	HLA-DQA1	NT1 (*n* = 40)	NT2 (*n* = 14)	Total (*n* = 54)
0602 homozygous	11 (19.6%)	3 (13.6%)	14 (17.9%)	0102 homozygous	12 (30%)	3 (21.4%)	15 (27.8%)
0602 heterozygous	45 (80.4%)	11 (50%)	56 (71.8%)	0102 heterozygou	28 (70%)	7 (50%)	35 (64.8%)
0202,0602	2 (3.6%)	2 (9.1%)	4 (5.1%)	0101,0102	1 (2.5%)		1 (1.9%)
0301,0602	7 (12.7%)	1 (4.5%)	8 (10.3%)	0102,0103	1 (2.5%)		1 (1.9%)
0303,0602	1 (1.8%)		1 (1.3%)	0102,0104	2 (5%)		2 (3.7%)
0401,0602	1 (1.8%)	1 (4.5%)	2 (2.6%)	0102,0201	5 (12.5%)	1 (7.1%)	6 (11.1%)
0402,0602	1 (1.8%)		1 (1.3%)	0102,0301	1 (2.5%)	1 (7.1%)	2 (3.7%)
0501,0602	1 (1.8%)		1 (1.3%)	0102,0302	5 (12.5%)		5 (9.3%)
0502,0602	2 (3.6%)		2 (2.6%)	0102,0503	1 (2.5%)	1 (7.1%)	2 (3.7%)
0503,0602	2 (3.6%)		2 (2.6%)	0102,0505	7 (17.5%)	1 (7.1%)	8 (14.8%)
0601,0602	4 (7.3%)		4 (5.1%)	0102,0508	1 (2.5%)		1 (1.9%)
0602,0202	5 (8.9%)		5 (6.4%)	0102,0601	4 (10%)	3 (21.4%)	7 (13%)
0602,0301	11 (19.6%)	4 (18.2%)	15 (19.2%)	0102 negative		4 (28.6%)	4 (7.4%)
0602,0302	1 (1.8%)	1(4.5%)	2 (2.6%)	0101,0201		1 (7.1%)	1 (1.9%)
0602,0303	5 (8.9%)		5 (6.4%)	0101,0302			
0602,0601	1 (1.8%)		1 (1.3%)	0103,0303		1 (7.1%)	1 (1.9%)
0602,0604		2(9.1%)	2 (2.6%)	0104,0601		1 (7.1%)	1 (1.9%)
0602,0609	1 (1.8%)		1 (1.3%)	0105,0505		1 (7.1%)	1 (1.9%)
0602 negative		8 (36.3%)	8 (10.3%)				
0202,0301		1 (4.5%)	1 (1.3%)				
0202,0303		1 (4.5%)	1 (1.3%)				
0302,0601		1 (4.5%)	1 (1.3%)				
0401,0502		1 (4.5%)	1 (1.3%)				
0501,0202		1 (4.5%)	1 (1.3%)				
0501,0301		1 (4.5%)	1 (1.3%)				
0503,0301		1 (4.5%)	1 (1.3%)				
0601,0401		1 (4.5%)	1 (1.3%)				

**Figure 1:**
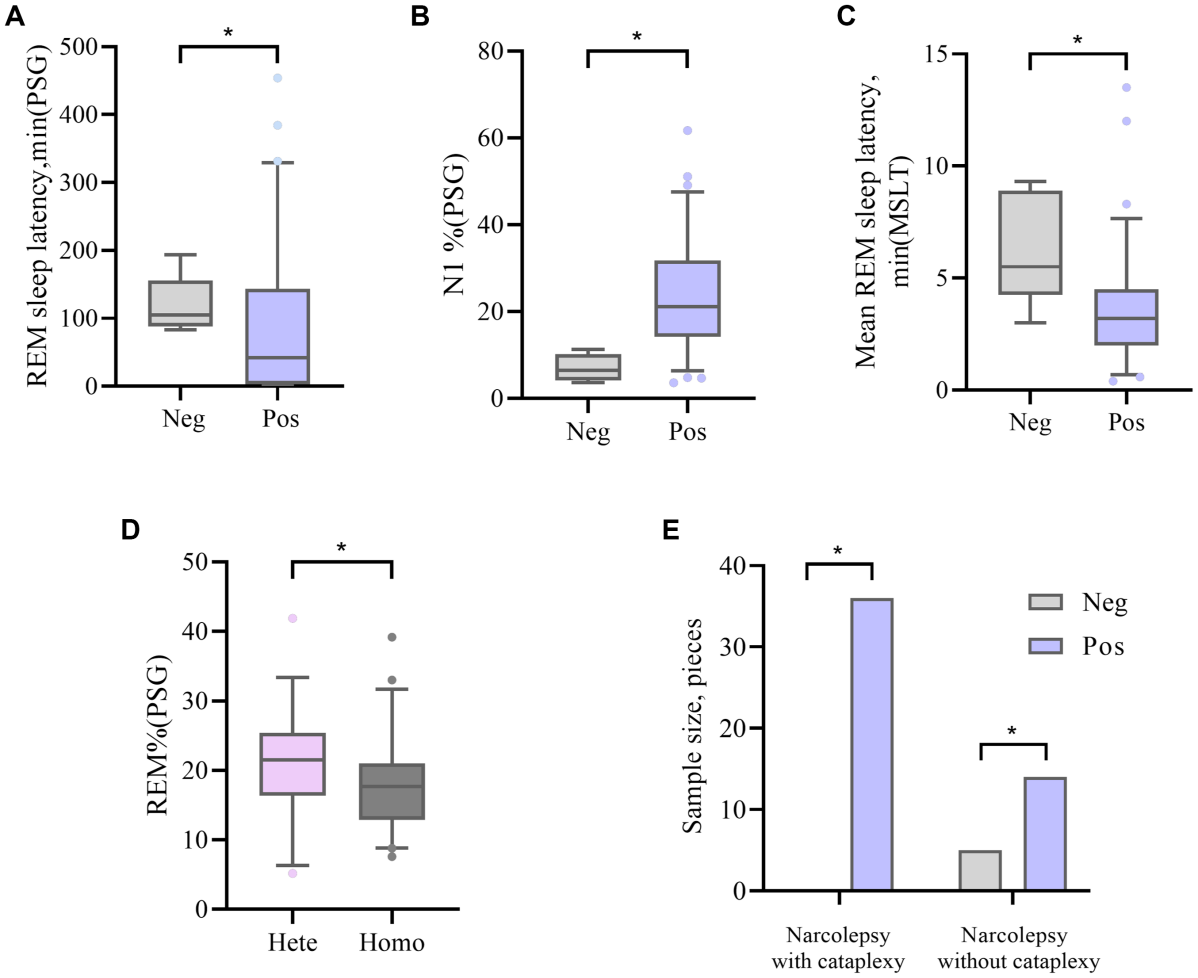
**(A, B)** The difference in REM sleep latency and N1% between DQA1*0102 Neg and DQA1*0102 Pos in PSG. **(C)** The difference in average REM sleep latency between DQA1*0102 Neg and DQA1*0102 Pos in MSLT. **(D)** The difference in REM% between DQA1 * 0102 Hete and DQA1 * 0102 Homo in PSG. **(E)** The difference in expression frequency of DQA1*0102 Neg and DQA1*0102 Pos in narcolepsy with and without cataplexy. Neg: negative; Pos: positive; Hete: Heterozygous; Homo: Homozygous; REM: rapid eye movement; N1%: the proportion of non rapid eye movement sleep phase 1; REM%: the proportion of sleep during the REM phase; PSG: Polysomnography; MSLT: Multiple sleep latency tests.

The HLA-DQA1*0102-positive and HLA-DQA1*0102-negative patients showed significant differences (*p* < 0.05) in disease course, presence or absence of sudden onset, REM sleep latency according to PSG, percentage of N1 sleep, and average REM latency according to the MSLT. DQA1*0102-positive patients had a longer disease course, were prone to entering REM sleep at night and during the day, and had an increased proportion of N1 sleep at night, with shallow sleep often accompanied by sudden onset. There was a statistically significant difference (*p* < 0.05) in the average REM latency in the MSLT between DQA1*0102 positive and negative patients, and there was no difference in basic information, appetite hormone levels, scale scores, or sleep parameters. A lower proportion of DQA1*0102 homozygous individuals experienced REM sleep at night (Table 3 and Figure 1).

**Table 3:** Differences in clinical and somatosensory characteristics among patients with narcolepsy with different HLA-DQA1*0102 genotypes.

	DQA1 *0102					
	Neg (*n* = 5)	Pos (*n* = 50)	*p*-value	Hete (*n* = 35)	Homo (*n* = 15)	*p*-value
Orexin-A, pg./ml	181.43 (150.78 ~ .)	20.02 (13.1425 ~ 59.6275)	ns	20.735 (14.3575 ~ 53.395)	17.815 (0 ~ 115.8225)	ns
Clinical features						
Cataplexy	1 (20%)	39 (78%)	<0.05*	29 (82.9%)	10 (66.7%)	ns
Sleep paralysis	2 (40%)	15 (30%)	ns	10 (28.6%)	5 (33.3%)	ns
Hypnagogic hallucinations	1 (20%)	17 (34%)	ns	9 (25.7%)	8 (53.3%)	ns
RBD	1 (20%)	16 (32%)	ns	10 (28.6%)	6 (40%)	ns
Scale rating						ns
PSQI	3 (2 ~ 6.5)	5 (3 ~ 7)	ns	5 (3 ~ 7)	6 (3 ~ 10)	ns
ESS	11 (7 ~ 17)	15 (10.5 ~ 19.75)	ns	15 (10 ~ 20)	15 (12 ~ 17)	ns
UNS	13 (9.5 ~ 22.5)	17 (11 ~ 24)	ns	15 (12 ~ 20)	21 (10 ~ 25)	ns
HAMA	8 (6 ~ 31.5)	8 (3 ~ 17)	ns	11 (3 ~ 17.75)	7 (4 ~ 13)	ns
HAMD	9 (3 ~ 9)	6 (2 ~ 9)	ns	6 (3.25 ~ 9)	4 (2 ~ 10)	ns
Nocturnal polysomnography						
Total sleep time, min	515 (407 ~ 524.75)	476.75 (418.8 ~ 518.1)	ns	485 (435.5 ~ 518)	464 (400 ~ 519)	ns
Sleep efficiency	93.6 (83.25 ~ 98.1)	89.55 (79.775 ~ 93.525)	ns	90 (81.1 ~ 93.9)	83.2 (73.8 ~ 92.3)	ns
Sleep latency, min	15 (4.25 ~ 29.75)	3 (1 ~ 8.75)	ns	3 (0.5 ~ 7)	4.5 (2 ~ 15)	ns
Mean REM sleep latency, min	105 (78.7 ~ 155.7)	6.75 (3 ~ 76.375)	<0.05*	5 (1.5 ~ 68.5)	12.5 (4.5 ~ 200)	ns
REM %	20.7 (17.1 ~ 28.5)	19.8 (15.375 ~ 24.375)	ns	21.5 (16.1 ~ 25.4)	16 (12.5 ~ 20.8)	<0.05*
N1%	6.4 (4.15 ~ 27.95)	20.4 (12.45 ~ 29.825)	<0.05*	20.4 (11.1 ~ 29.6)	18.3 (12.9 ~ 32.3)	ns
N2%	43.2 (34.5 ~ 51.7)	43.45 (33.15 ~ 49.45)	ns	42.5 (33.2 ~ 47.8)	46.4 (33 ~ 51.5)	ns
N3%	17.5 (8.15 ~ 34.35)	16(11.45 ~ 21.3)	ns	17.1 (11.3 ~ 21.3)	15.1 (11.5 ~ 21.3)	ns
AHI	1.7 (0.3 ~ 10.3)	3.1 (0.5 ~ 7.6)	ns	3.55 (0.5 ~ 6.925)	1.4 (0.3 ~ 11.8)	ns
Oxygen reduction index	0.7 (0.25 ~ 5.35)	2.3 (0.55 ~ 4.55)	ns	2.3 (0.575 ~ 4.05)	3.2 (0.4 ~ 11.4)	ns
Micro arousal index	5.5 (2.8 ~ 17.9)	11.15 (5.375 ~ 16.225)	ns	11.2 (5 ~ 15.6)	10.9 (5.5 ~ 20.1)	ns
Multiple sleep latency test						
MSL, min	4.6 (2.2 ~ 6.9)	3.1 (1.95 ~ 5.45)	ns	2.95 (1.8 ~ 4.6)	3.6 (2.3 ~ 8.3)	ns
Mean REM sleep latency, min	5.5 (3.2 ~ 8.9)	3 (1.95 ~ 4.4)	<0.05*	3.2 (2.2 ~ 4.85)	2.2 (1.4 ~ 3.45)	ns
The number of times SOREMPS	3 (1.5 ~ 4)	4 (3 ~ 5)	ns	4 (3 ~ 5)	4 (2 ~ 5)	ns

The categorical variables are described using Fisher’s exact test using “N, %,” and the continuous variables are represented using the Mann–Whitney U test using “median (range).” Pos, positive; Neg, negative; Hete, heterozygosity; Homo, homozygosity; REM, rapid Eye movement; MSL, mean sleep latency; SOREMP, sleep-onset REM period; AHI, apnea-hypopnea index; N1, non-rapid Eye movement phase 1 sleep.

**Figure 2:**
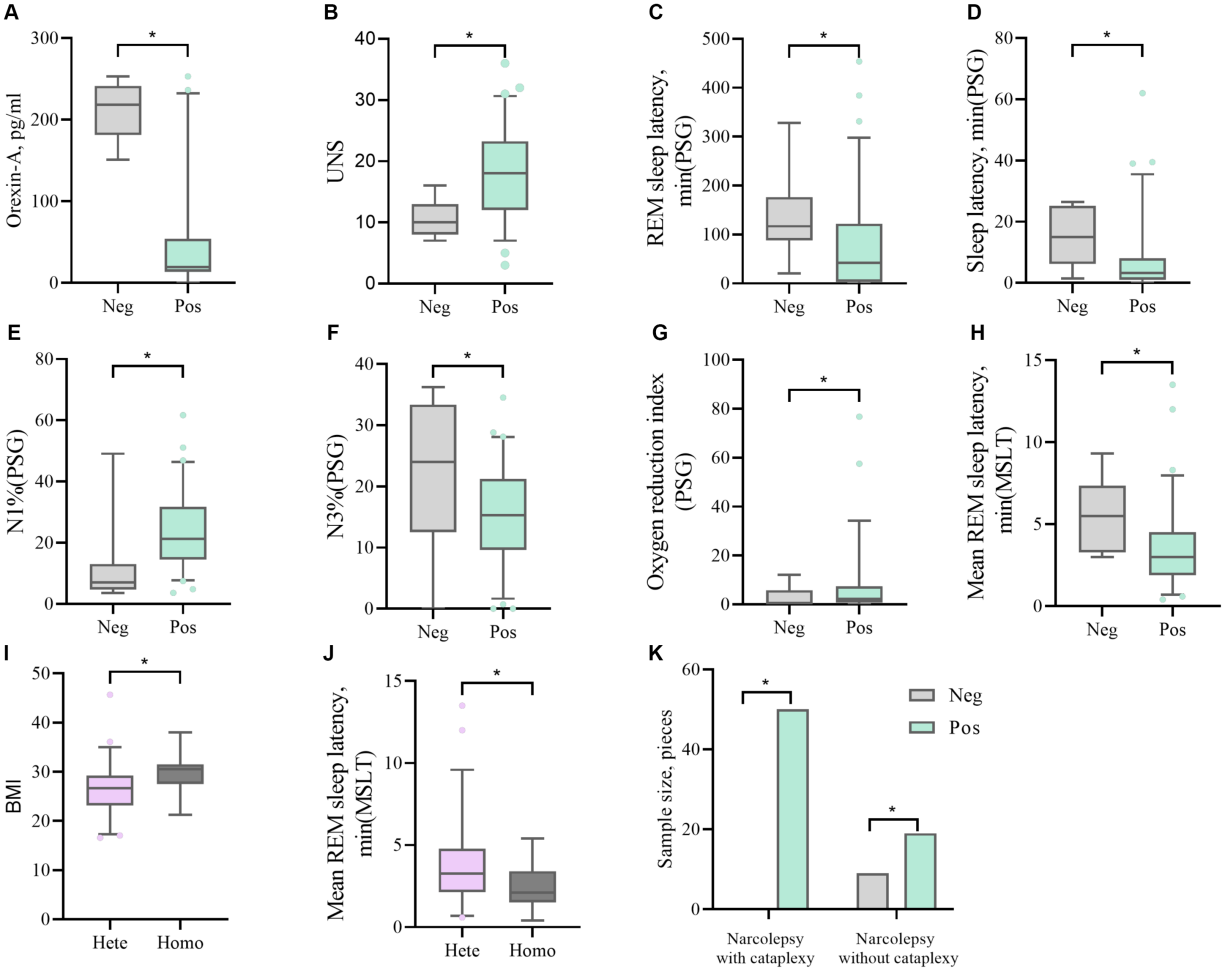
**(A, B)** The difference in Orexin-A levels and UNS scores between DQB1*0602 Neg and DQB1*0602 Pos. **(C, D, E, F, G)** Differences in REM sleep latency, sleep latency, N1%, N3%, and oxygen depletion index between DQB1*0602 Neg and DQB1*0602 Pos in PSG. **(H)** The difference in average REM sleep latency between DQB1*0602 neg and DQB1*0602 Pos in MSLT. **(I)** The difference in BMI between DQB1*0602 Hete and DQB1*0602 Homo. **(J)** The difference in average REM sleep latency between DQB1 * 0602 Hete and DQB1 * 0602 Homo in MSLT. **(K)** The difference in expression frequency of DQB1*0602 Neg and DQB1*0602 Pos in narcolepsy with and without cataplexy. Neg: negative; Pos: positive; Hete: Heterozygous; Homo: Homozygous; REM: rapid eye movement; N1%: the proportion of non rapid eye movement sleep phase 1; N3%: the proportion of non rapid eye movement sleep phase 3; PSG: Polysomnography; MSLT: Multiple sleep latency tests; UNS: Ullanlinna Narcolepsy Scale; BMI: body mass index.

There were significant differences (*p* < 0.05) between HLA-DQB1*0602-positive and HLA-DQB1*0602-negative patients in terms of orexin-A levels, the presence or absence of sudden onset, sleep latency in the UNS and PSG, REM sleep latency, percentage of N1 sleep, the oxygen depletion index, and average REM latency in the MSLT. DQB1*0602-positive patients had significantly lower orexin-A levels, greater UNS, significantly shorter nighttime sleep latency and REM sleep latency, an increased proportion of N1 sleep, and a decreased proportion of N3 sleep. Moreover, REM sleep easily occurs during the day, and there was a statistically significant difference (*p* < 0.05) in the average REM latency between homozygous and heterozygous patients with MSLT in DQB1*0602. There were no differences in basic data, orexin-A levels, scale scores, or other sleep parameters. Compared with heterozygous individuals, homozygous individuals with DQB1*0602 had a greater BMI and shorter average REM latency during naps (Table 4 and Figure 2).

**Table 4:** Differences in clinical and somatosensory characteristics among patients with narcolepsy with different HLA-DQB1*0602 genotypes.

	DQB1*0602					
	Neg (*n* = 8)	Pos (*n* = 70)	*p*-value	Hete (*n* = 57)	Homo (*n* = 13)	*p*-value
Orexin-A, pg./ml	218.31 (181.43 ~ 241.305)	19.595 (13.5125 ~ 54.09)	<0.01**	20.02 (14.295 ~ 54.01)	13.59 (0 ~ 106.85)	ns
Clinical features						
Cataplexy	1 (12.5%)	55 (78.6%)	<0.01**	45 (78.9%)	10 (76.9%)	ns
Sleep paralysis	1 (12.5%)	55 (78.6%)	ns	45 (78.9%)	10 (76.9%)	ns
Hypnagogic hallucinations	2 (25%)	23 (32.9%)	ns	17 (29.8%)	6 (46.2%)	ns
RBD	3 (37.5%)	19 (27.1%)	ns	16 (28.1%)	3 (23.1%)	ns
Scale rating						
PSQI	4 (3 ~ 7.5)	5 (3 ~ 7)	ns	5 (3 ~ 7)	7 (3.5 ~ 9.5)	ns
ESS	12.5 (11 ~ 16)	16 (14 ~ 19)	ns	16 (14 ~ 19.25)	15 (11 ~ 16)	ns
UNS	10 (8 ~ 13)	18 (12 ~ 23.25)	<0.01**	18 (12.5 ~ 23)	16 (10.5 ~ 25)	ns
HAMA	11 (5.5 ~ 28.75)	8 (3.75 ~ 16.25)	ns	8 (3 ~ 17)	7 (4.5 ~ 14.5)	ns
HAMD	9 (2.5 ~ 11.25)	5 (3 ~ 8.5)	ns	6 (2.25 ~ 8.75)	5 (3 ~ 9)	ns
Nocturnal polysomnography						
Total sleep time, min	512.5 (431.25 ~ 525.375)	469 (397.125 ~ 515)	ns	469 (404.25 ~ 516)	462 (328.25 ~ 515.75)	ns
Sleep efficiency	92.6 (87.75 ~ 96.475)	88.55 (77.525 ~ 93.175)	ns	89.1 (76.55 ~ 93.25)	83.2 (74.05 ~ 93.45)	ns
Sleep latency, min	17.25 (5.875 ~ 25.875)	3.4 (1 ~ 8.125)	<0.05*	3.3 (1.25 ~ 7.5)	4 (0.75 ~ 16.25)	ns
Mean REM sleep latency, min	117.5 (88.5 ~ 185)	42 (3 ~ 136.75)	<0.05*	42 (3 ~ 104.25)	55 (3.75 ~ 179.75)	ns
REM %	17.1 (11.425 ~ 19.95)	19.5 (15 ~ 23.4)	ns	19.4 (13.95 ~ 23.4)	19.6 (15.6 ~ 22.85)	ns
N1%	6.7 (4.7 ~ 13.325)	21.2 (14.375 ~ 31.925)	<0.01**	21.3 (14.25 ~ 32.65)	20.8 (12.65 ~ 32.05)	ns
N2%	43.5 (42.825 ~ 53.65)	42.7 (32.675 ~ 51.425)	ns	42.5 (32.45 ~ 50.15)	43.9 (31.95 ~ 51.95)	ns
N3%	24.4 (13.975 ~ 33.775)	15.25 (9.575 ~ 21.225)	<0.05*	15.1 (9.3 ~ 20.55)	19 (13.3 ~ 22.25)	ns
AHI	1.75 (0.225 ~ 8.4)	3.3 (0.6 ~ 11.45)	ns	3.55 (0.9 ~ 11.625)	1.2 (0.2 ~ 11.5)	ns
Oxygen reduction index	0.55 (0.125 ~ 4.15)	2.3 (0.75 ~ 8.7)	<0.05*	2.35 (0.725 ~ 9.2)	1.3 (0.75 ~ 7.35)	ns
Micro arousal index	5.75 (3.25 ~ 17.3)	11 (6.875 ~ 15.75)	ns	11.2 (6.85 ~ 16.4)	9.7 (6.25 ~ 11.75)	ns
Multiple sleep latency test						
MSL, min	6.9 (2.875 ~ 11.25)	3 (1.8 ~ 5.35)	ns	2.95 (1.825 ~ 4.6)	3.6 (1.8 ~ 8.9)	ns
Mean REM sleep latency, min	4.85 (3.3 ~ 7.925)	3.15 (2.025 ~ 4.65)	<0.05*	3.4 (2.2 ~ 4.85)	2.1 (1.5 ~ 3.4)	<0.05*
The number of times SOREMPS	3 (2 ~ 4.75)	4 (3 ~ 5)	ns	4 (3 ~ 5)	4 (3 ~ 5)	ns

The categorical variables are described using Fisher’s exact test using “*N*, %,” and the continuous variables are represented using the Mann–Whitney U test using “median (range).” Pos, positive; Neg, negative; Hete, heterozygosity; Homo, homozygous; REM, rapid Eye movement; MSL, mean sleep latency; SOREMP, sleep-onset REM period; AHI, apnea-hypopnea index; N1, nonrapid Eye movement phase 1 sleep.

## Discussion

Narcolepsy is a central sleep disorder characterized by excessive daytime sleepiness, cataplexy, hypnagogic hallucinations, sleep paralysis, and nighttime sleep disturbances. Narcolepsy with cataplexy, named narcolepsy type 1, is caused by a deficiency in the hypothalamic neuropeptide orexin; the exact cause is not fully understood, but narcolepsy type 1 is generally believed to be an autoimmune reaction caused by the interaction of environmental and genetic factors (1, 3).

This study revealed that the homozygosity of HLA-DQA1/DQB1 is related to susceptibility and sleepiness. We observed that the most common susceptibility genes in patients with narcolepsy in this study were HLA-DQB1*0602/*0301/HLA-DQA1*0102/*0505. The expression of HLA-DQB1*0301 even exceeded that of HLA-DQB1*0602. HLA-DQA1*0102/DQB1*0602 gene-positive individuals accounted for 91% of all patients, which is consistent with the findings of F Han’s research and represents the genotypic characteristics of Chinese people (15). We conducted a statistical analysis of dozens of relevant articles published in PubMed and the China National Knowledge Infrastructure (CNKI). Among 4,000 healthy Han Chinese individuals, the average frequency of HLA-DQA1*0102 expression was 20%, and the average frequency of HLA-DQB1*0602 expression was 23%, which was significantly different from the gene expression frequency in narcoleptic patients. At present, there are already published reports on large-scale HLA-A, HLA-B, HLA-C, DQA1, and DQB1 gene polymorphism data in China (21, 22), and a table of common and determined alleles suitable for the Chinese population has been established. Our study mainly analyzed the relationships between different genotypes and clinical symptoms of narcolepsy in northern China.

Second, the different genotypes of HLA-DQA1*0102/DQB1*0602 are closely related to the presence or absence of cataplexy but not to hallucinations, sleep paralysis, or RBD. A total of 78% of HLA-DQA1*0102/DQB1*0602-positive patients had type 1 narcolepsy. Moreover, heterozygous individuals have overall low levels of orexin, indicating a clear relationship between this gene and type 1 narcolepsy or that patients with and without cataplexy have different pathogeneses (23). NT1, NT2, and idiopathic hypersomnia (IH) are the three main central somnolence diseases and are mainly distinguished by clinical symptoms, PSG, MSLT, and hypothalamic orexin levels. NT1 has clear biomarkers, while the diagnostic boundaries of NT2 and IH are relatively vague, and the classification problem is still controversial (24). Capitini et al. conducted a meta-analysis (25) to evaluate the effectiveness of HLA testing in the diagnosis of narcolepsy and idiopathic drowsiness in four major racial groups (Asians, African Americans, Native Americans, and Caucasians). A total of 2077 patients with NT1, 235 patients with NT2, 161 patients with idiopathic lethargy, and 7,802 controls were included in the study. HLA-DQB1*0602 carriers had a 5-fold increased risk of sudden death, and there was a correlation with the development of NT2, which was slightly lower than that of NT1. Our data also suggest that two-thirds of NT2 patients carry genetic susceptibility genes, with two-thirds of patients testing for slightly abnormal or normal levels of orexin-A and the remaining one-third refusing lumbar puncture surgery. Because NT2 is still an undefined disease, from a clinical perspective, patients with positive HLA DQB1*0602 in the NT2 population should be encouraged to test for hypothalamic secretions, potentially indicating insufficient appetite. IH is not related to HLA DQB1*0602. Therefore, HLA typing can serve as a complementary diagnostic marker with the potential to improve sensitivity in diagnosing different types (NT1, NT2, and IH).

The homozygosity of DQA1*0102-DQB1*0602 leads to an increase in the number of susceptible dimers expressed, supporting the dose effect on etiology (9). This phenomenon is mainly due to the 1.65-fold greater mRNA gene expression and 1.59-fold greater B-cell surface protein levels in DQB1*0602 homozygous individuals than in heterozygous individuals, resulting in a 2- to 3-fold greater risk of narcolepsy episodes in DQB1*0602 homozygous individuals than in heterozygous individuals (26). In 1998, Pelin (27) and others conducted a similar study and reported that HLA-DQB1*0602 homozygosity increases susceptibility to narcolepsy but does not seem to affect the severity of the disease. We also found that there was no difference in symptoms between HLA-DQA1*0102/DQB1*0602 homozygous individuals and other individuals. However, the average REM latency in the MSLT was shortened, and patients entered the REM phase more quickly. The degree of drowsiness was relatively severe. However, it should be comprehensively judged based on the average MSLT sleep latency, PSG sleep latency, ESS score, and other factors. We believe that the reasons for these different results may also be related to race. Pelin et al. (27) mainly analyzed African Americans and Caucasian Americans. MSLT is a necessary condition for diagnosing narcolepsy. When the average REM latency was ≤5 min (sensitivity/specificity/positive predictive value: 49%/95%/96%), the best differentiation effect between narcolepsy and nonnarcolepsy patients was achieved. In the past 20 years, researchers have found that SOREMPs are also present in individuals with various high sleep stress disorders (28), such as sleep deprivation syndrome, shift syndrome, sleep phase delay syndrome, sleep breathing disorders, Parkinson’s syndrome, depression, and other psychiatric disorders.

In this study, we found that HLA-DQA1*0505 expression was second only to HLA-DQA1*0102 heterozygosity. It is known that HLA-DQA1*0505 is a protective gene for type 1 diabetes (29) and breast cancer (30) and is also a susceptibility factor for common variant immunodeficiency (31) and chronic myeloid leukemia (32). It is also closely related to the elevation of blood pressure in autoimmune blister disease (33) and measles-specific IL-12p40 secretion (34) and may be involved in the regulation of specific immune responses to common allergens (35). There is currently no relevant research confirming the correlation between this gene and narcolepsy, and a larger sample size is needed for analysis.

Additionally, in this study we observed that a small proportion of patients with narcolepsy did not carry HLA alleles, indicating that HLA alleles themselves cannot fully explain the mechanism of narcolepsy. In cohort studies in Japan and South Korea, GWAS was used to identify a non-HLA-related variant located between CHKB and CPT1B that is significantly associated with narcolepsy (36). A variant of the T-cell receptor site α (TCRA) was found to be significantly associated with sudden onset encephalopathy in a mixed European and Asian lineage cohort study (37). Two other studies have also found non-HLA risk gene loci associated with narcolepsy, including CTSH and TNFSF4 (38), P2RY11, TCRB, IL10RB-INFAR1, and ZNF365 (39). Professor Han Fang conducted a study on 903 patients with narcolepsy and 1981 healthy controls, evaluating the relationship between 32 previously reported SNPs related to susceptibility to narcolepsy and the risk of drowsiness. GRS4 is most closely related to the risk of drowsiness in the Chinese population. A significant correlation between the GRS score and the risk of episodic sleep, which can promote risk stratification prevention trials for HLA-DQB1*0602-positive and HLA-DQB1*0602-negative individuals (40).

In summary, this study demonstrated a correlation between different genotypes of HLA-DQA1*0102/DQB1*0602 and cataplexy in Chinese patients with narcolepsy. Homozygous individuals in the MSLT have a shorter average REM latency, greater genetic susceptibility, and relatively greater drowsiness. However, this was a single-center study with a small sample size, and there may be selection bias as a result. In the future, the sample size will be further expanded, stratified analysis will be conducted on different genotypes, and further correlation analysis will be conducted on neuropsychological tests and sleep structural parameters to further explore the genetic mechanism of narcolepsy.

## Data availability statement

The original contributions presented in the study are included in the article/supplementary material, further inquiries can be directed to the corresponding author.

## Ethics statement

The studies involving humans were approved by the Human Research Ethics Committee of The First Affiliated Hospital of Shandong First Medical University. The studies were conducted in accordance with the local legislation and institutional requirements. Written informed consent for participation in this study was provided by the participants’ legal guardians/next of kin.

## Author contributions

WZ: Data curation, Writing – original draft. BZ: Investigation, Writing – review & editing. ZY: Visualization, Writing – review & editing. MZ: Data curation, Writing – review & editing. XZ: Writing – review & editing. XYZ: Writing – review & editing. XL: Writing – review & editing. JT: Methodology, Writing – review & editing.
